# Complete Genome of *Acinetobacter baumannii* Podophage Petty

**DOI:** 10.1128/genomeA.00850-13

**Published:** 2013-12-05

**Authors:** Ian P. Mumm, Thammajun L. Wood, Karthik R. Chamakura, Gabriel F. Kuty Everett

**Affiliations:** Center for Phage Technology, Texas A&M University, College Station, Texas, USAa; Department of Biochemistry and Molecular Biology, Pennsylvania State University, University Park, Pennsylvania, USAb

## Abstract

*Acinetobacter baumannii* is an emerging pathogen that was isolated from wounded soldiers in military treatment facilities in Iraq but has since become a problem in civilian hospitals. Here, we announce and describe the complete genome of the ϕKMV-like *A. baumannii* podophage Petty.

## GENOME ANNOUNCEMENT

*Acinetobacter baumannii* is a multidrug-resistant emerging pathogen. It has been nicknamed “Iraqibacter” because of its origin in military hospitals in Iraq and its persistence among veterans (1, 2). There is increasing interest in alternative methods, such as bacteriophage therapy, to control this pathogen. Here, we present the genome of *A. baumannii* phage Petty.

Bacteriophage Petty was isolated from a sewage sample collected in College Station, TX. Phage DNA was sequenced using 454 pyrosequencing at the Emory GRA Genome Center (Emory University, Atlanta, GA). The trimmed FLX Titanium reads were assembled to a single contig at 88.4-fold coverage using the Newbler assembler version 2.5.3 (454 Life Sciences) with the default settings. The contig was confirmed to be complete by PCR. Genes were predicted using GeneMarkS (3) and corrected using software tools available on the Center for Phage Technology (CPT) portal (https://cpt.tamu.edu/cpt-software/portal/).

Petty is a ϕKMV-like podophage (4). Its unit genome has 40,431 bp, a G+C content of 42.3%, a coding density of 92.2%, and 45 coding sequences. The processing of raw pyrosequencing reads using the Pause method (https://cpt.tamu.edu/cpt-software/releases/pause/) revealed terminal repeats of 308 bp in length, twice the size of the repeats in phage T7 and 106 bp shorter than the repeats seen in ϕKMV.

Petty was found to have clusters of genes encoding proteins corresponding to certain processes, such as DNA replication and viral assembly. The left end of the genome is occupied by novel and conserved genes of unknown function. The conserved genes are homologs of genes from the ϕKMV-like *A. baumannii* phage ϕAB1 (5). The next group of genes encodes replication and recombination proteins, such as DNA primase, helicase, ligase, DNA polymerase, and several nucleases. Petty encodes one DDE endonuclease, in contrast to the four HNH homing endonucleases in ϕAB1. The carboxylic acid residues of the DDE motif in this superfamily of endonucleases coordinate a metal ion needed for catalysis (6). Like phages T7 and T3, Petty encodes its own RNA polymerase (7). As with other ϕKMV-like phages, the RNA polymerase gene of Petty is adjacent to the morphogenesis genes rather than to the early genes like in T7 and T3 (8, 9). The last gene cluster includes genes encoding proteins for morphogenesis, lysis, and DNA packaging. The morphogenesis proteins include head-to-tail joining protein, scaffold protein, capsid protein, tail tube subunits, internal core proteins, and a T7-like tail fiber protein (InterPro database entry IPR005604). The lysis genes include genes for a class II holin and an endolysin (identified by its peptidoglycan binding domain and its position next to the holin). Strikingly, the lysis cassette does not include spanin genes. Spanins disrupt the outer membrane and are important for lysis in Gram-negative hosts (10). There are two types of spanins, two component spanins and unimolecular spanins. In both cases, one of the spanin proteins is an outer membrane lipoprotein, and Petty encodes no lipoproteins. This absence suggests that Petty causes lysis by a novel mechanism, at least for outer membrane disruption.

### Nucleotide sequence accession number.

The genome of phage Petty was contributed as accession no. KF669656 to GenBank.
